# Progress in the risk assessment of hydroxychloroquine in frail elderly people

**DOI:** 10.1002/agm2.12140

**Published:** 2020-12-25

**Authors:** Alberto Castagna, Giovanni Ruotolo, Ciro Manzo

**Affiliations:** ^1^ Azienda Sanitaria Provinciale Catanzaro, Primary Care Departiment, Center for Cognitive Disorders and Dementia Catanzaro Italy; ^2^ Geriatric Unit General Hospital Azienda Ospedaliera Pugliese‐Ciaccio di Catanzaro Catanzaro Italy; ^3^ Azienda Sanitaria Locale Napoli 3 Sud Internal and Geriatric Medicine Department ‐ Gerontorheumatological Outpatient Clinic Poliambulatorio “Mariano Lauro” ‐ Distretto Sanitario 59 Naples Italy

**Keywords:** COVID‐19, hydroxychloroquine, QTc prolongation risk score

## Abstract

Hydroxychloroquine (HCQ) is an antimalarial drug also known to have anti‐inflammatory and antiviral effects. The antiviral action of HCQ has been a point of interest for many researchers because of its mechanism of action and the potential use it could have during the current COVID‐19 pandemic. However, HCQ can cause QT interval prolongation. The current therapies used in COVID‐19 are changing as the pandemic develops. The aim of this article is to promote a validated risk score for QT prolongation in multidimensional assessment of COVID‐19 patients, especially in elderly and polypathological patients.

## Graphical abstract

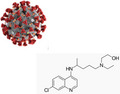

## 1 INTRODUCTION

COVID‐19 has been declared a global pandemic by the World Health Organization^1^. Currently, we are lacking therapeutic options.^2^ Some observational studies have suggested the benefits of hydroxychloroquine (HCQ) for the treatment of COVID‐19.^2^ As a result, these treatments are increasingly used off‐label for patients with COVID‐19. Over the past few decades, HCQ has received wide attention as an antiviral drug. Wang et al^3^ in 2014 revealed that HCQ activates the host antiviral innate immunity. This drug accumulates in the cellular organelles, creating an acidic environment and inhibiting the replication of different viruses by interfering with endosome/lysosome trafficking or viral protein maturation.^4^ Clinical trials have revealed that HCQ is able to act as a potential drug in fighting against severe acute respiratory syndrome‐coronavirus 2 (SARS‐CoV‐2).^5^, ^6^, ^7^ HCQ is structurally and mechanistically similar to the class IA antiarrhythmic quinidine, which inhibits voltage‐gated sodium and potassium channels, prolonging the QT interval and increasing the risk of torsades de pointes (TdP) and sudden cardiac death.^8^ Chloroquine’s reported risk of sudden cardiac death is limited to cases of hypotension due to vasodilation and negative inotropy resulting from rapid parenteral administration of the medication or situations of self‐inflicted overdose.^9^ The risk of QT prolongation and TdP with HCQ is limited to a series of case reports in patients with either chronic use or overdose.^10^ In patients treated with HCQ and azithromycin, Chorin et al observed prolongation of the corrected QT interval (QTc) from a baseline average of 435 ± 24 ms (mean ± SD) to a maximal average value of 463 ± 32 ms (*P* < .001 [one‐sample *t*‐test]), which occurred on Day 3.6 ± 1.6 of therapy.^11^ Mercuro et al observed that patients who received HCQ for the treatment of pneumonia associated with COVID‐19 were at high risk of QTc prolongation, and concurrent treatment with azithromycin was associated with greater changes in QTc.^12^

Large population data from the US National Health and Nutrition Examination Survey II and III studies reported that heart‐rate‐QTc variance on an electrocardiogram (ECG) increases with age, and specifically, prolonged QTc is more prevalent in older individuals.^13^ In addition, prolonged QTc is associated with an increased risk of incident cardiovascular event or cardiovascular death.^14^ The QT interval is an indirect measure of the duration of ventricular depolarization and repolarisation.^15^ A prolongation of the QT interval and an increased QTc are associated with an increased rate of cardiovascular morbidity and mortality. This is related to electrical instability and the risk of ventricular arrhythmogenesis.^16^, ^17^ QTc increases with age may be due to a combination of factors. Aging is associated with alterations in the amount of sympathetic and parasympathetic tone that can alter myocardial repolarization and the duration of the QTc.^18^, ^19^ The relationship between advancing age and QT interval might be explained by the changes in heart and vasculature observed in elderly subjects. These include cardiac hypertrophy, increased vascular stiffness, and aortic impedance. The increase in vascular impedance, in conjunction with its effects leading to an increase in systolic arterial pressure, means that the pulsate component of external cardiac work must increase.^20^ The stroke work, expressed as the product of stroke volume and systolic blood pressure, has been shown to increase with age in clinically normotensive subjects. This occurs even in elderly subjects in whom stroke volume at rest is decreased compared to younger subjects.^21^ In addition, traditional cardiovascular disease risk markers gradually lose their predictive value with age.^22^, ^23^ The main reason for this high prevalence is that Italy is the second oldest nation in the world. In fact, like other pathological conditions common in elderly patients, the mortality rate of COVID‐19 is directly related to the patient’s age, frailty, and comorbidities.^24^ Finally, recent data suggest that frailty‐related phenotypes are associated with QTc prolongation. Michishita et al reported that inactivity and light‐intensity physical activity were associated with QTc in older adults.^25^ The analysis of the data collected with the Comprehensive Geriatric Assessment is particularly useful and effective in the management of geriatric symptoms and syndromes, especially in situations of complexity/emergency, the recent COVID‐19 pandemic among these.^26^

Pre‐existing cardiovascular diseases are widely represented in these patients and are associated with a poorer prognosis.^24^ Beyond the acquired association between pre‐existing cardiovascular diseases and the severity of respiratory infections with negative outcome for patients, attention should be focused on cardiovascular complications directly associated with COVID‐19. Very often in COVID‐19 patients, especially in severe forms evolving into acute respiratory distress syndrome, cardiac injury appears with increased troponin values,^27^, ^28^ which are not necessarily associated with an acute coronary syndrome, as they can also be related to non‐ischemic forms, such as myocarditis.^29^ The destabilization of a pre‐existing coronary plaque, mediated by the systemic inflammatory process with consequent rupture of the fibrous cap, exposure of thrombogenic material, and thrombotic occlusion of the vessel, represents the most probable pathogenetic hypothesis. The inflammatory state contributes to this sequence of events through several determinants, such as the release of inflammatory cytokines, sympathetic hyperactivation, increased free radicals and wall stress, tachycardia, hypoxia, and finally a state of increased thrombophilia.^24^ However, HCQ can cause QT interval prolongation. For this reason, we need to evaluate its safety profile, especially in polytherapy elderly patients. The normal QTc in adults is 0.36 to 0.47 seconds (360‐470 ms) in males and 0.36 to 0.48 seconds (360‐480 ms) in females.^29^ Different medications, including drugs prescribed for non‐cardiac indications, can cause QT interval prolongation (defined as ≥470 ms in males and ≥480 ms in females), and trigger TdP, which may degenerate into ventricular fibrillation and may result in unexpected cardiac arrest.^30^, ^31^ In the study presented by Molina et al,^32^ a single patient treated with HCQ showed ECG evidence of QT prolongation. Chorin et al^33^ observed that in around 11% of the population treated with the combination therapy, HCQ plus azithromycin showed evidence of significant QTc prolongation (>500 ms). In this medical case, the development of acute renal failure is an important predictor of extreme QTc prolongation.^29^ Thus, HCQ needs adjustment based on renal function: 200 mg × 2/d if estimated glomerular filtration rate (eGFR) >30 mL/min; 200 mg/d if 15 <eGFR<30 mL/min; 200 mg every other day if <15 mL/min (or in 3‐weekly or bi‐weekly dialysis).^34^, ^35^

QT interval prolongation is recognized as an ECG sign that portends an increased risk for TdP.^36^ This specific risk increases as the QTc increases.^37^, ^38^ Further, the risk of TdP related to a drug may be greater in polytherapy patients. Indeed, polytherapy patients show other risk factors, such as heart disease, advanced age, electrolyte abnormalities, bradycardia, and kidney or liver disease.^35^, ^39^, ^40^ A significant number of hospitalized patients with QTc prolongation receive QT‐interval‐prolonging drugs, which increase the risk of arrhythmia.^40^ As a consequence, identifying patients with highest risk of drug‐induced QTc prolongation could facilitate measures to modify the risk. The problem could be solved by giving discontinuously QT‐interval prolonging drugs, when possible.

## 2 QTc PROLONGATION RISK SCORE AND HCQ

Tisdale et al found a valid method to quantify the risk of drug‐induced QTc prolongation.^41^ The QTc prolongation risk score could be incorporated into multidimensional assessment to alert clinicians if the patient reveals a risk of drug‐induced QTc prolongation. The evaluation of QTc prolongation risk score includes the QTc‐prolonging value because the drugs are considered as a risk factor. Among the drugs able to prolong the QT interval we can mention anesthetic, antiarrhythmic, antidepressant, anticancer, antiemetic, antifungal, antimalarial, antipsychotic, and antibiotic medications.^42^ Their classifications are preferably based on the degree of QTc prolongation they induce: three points for taking one QTc‐prolonging drug and three additional points for taking ≥2 QTc‐prolonging drugs (for a total of six points). Later, the score could be classified as low risk (≤6 points), moderate risk (7‐10 points), or high risk (≥11 points). Caution is advised when combining QT‐prolonging medications or when using these medications in patients with electrolyte abnormalities.^43^

In the elderly patient in polytherapy, use of HCQ must be considered in relation to other risk factors. For instance, we have to evaluate how drugs included in polytherapy interact with HCQ metabolism. Indeed, HCQ is substrate for cytochrome P450 (CYP) enzymes (enzymes responsible for the metabolism of many drugs) and HCQ can interfere with other drugs.^44^, ^45^

Finally, smoking has previously been suspected to interfere with the bioavailability of HCQ.^45^

The findings from Mehra^46^ and colleagues’ study add to preliminary reports suggesting that regimens of chloroquine or HCQ , alone or with azithromycin, are not useful and could be harmful in hospitalized patients with COVID‐19.^47^ Boulware et al^48^ reported the results of a randomized trial testing HCQ as post‐exposure prophylaxis for COVID‐19, but the results are more provocative than definitive, suggesting that the potential prevention benefits of HCQ remain to be determined.^49^ This element could lead clinicians to select lower‐risk drug therapy, apply more intense QTc monitoring in susceptible patients, or implement other interventions to mitigate the risk (such as closer monitoring of serum electrolyte concentrations). On ClinicalTrials.gov, a total of 2,935 studies related to COVID‐19 were registered on August 7, 2020. A minority of clinical trials related to COVID‐19 registered on ClinicalTrials.gov planned to evaluate cardiovascular therapies and were estimated for completion after December 2020. Collectively, these findings underscore the need for a network of sites with a platform protocol for rapid evaluation of multiple therapies and generalizability to inform clinical care and health policy for COVID‐19 moving forward.^50^ The worldwide use of HCQ, beyond COVID‐19, requires a careful analysis of the risk factors for QTc prolongation in order to reduce the resulting arrhythmic risk. However, the simultaneous presence of several risk factors, very common in frail elderly patients with comorbidities, suggests the use of a multifactorial analysis model, in addition to the traditional comprehensive geriatric assessment.^51^

## 3 CONCLUSIONS

In our opinion, in clinical practice, the use of HCQ in the frail elderly must be used in a prudent way. ECG control is fundamental given the unpredictability, and it is prudent to correct electrolyte disorders and, where possible, avoid or minimize use of other drugs known to prolong the QT interval in relation to comorbidity and polypharmacotherapy.^52^

The current therapies used in COVID‐19 are changing according to the developing pandemic. Further study of the need for QT interval monitoring is needed before final recommendations can be made. Therefore, our article has been created to describe how multidimensional assessment, combined with a valuation of drug‐interaction risk in polypathological patients, remains a priority in the therapeutic management of all patients, including those with COVID‐19.

## CONFLICTS OF INTEREST

Nothing to disclose.

## AUTHOR CONTRIBUTIONS

Conceptualization: A.C., C.M., and G.R. Data curation: A.C. and G.R. Formal analysis: A.C. and C.M. Writing and original draft preparation: A.C., G.R., and C.M. Writing, review, and editing: A.C. and C.M. Supervision: A.C., C.M., and G.R. All authors agreed on the final text.
